# From Traits to Clusters: Emotional–Sensory–Regulatory Eating Profiles in Generation Z with Implications in Sustainable Food Behavior

**DOI:** 10.3390/nu18050758

**Published:** 2026-02-26

**Authors:** Maria P. Koliou, Amalia Kouskoura, Achilleas Kontogeorgos, Dimitris Skalkos

**Affiliations:** 1 Laboratory of Food Chemistry, Department of Chemistry, University of Ioannina, 45110 Ioannina, Greece; m.koliou@uoi.gr; 2 Management of Technology Research Lab (Materlab), University of Western Macedonia, 50100 Kozani, Greece; akouskoura@uowm.gr; 3 Department of Agriculture, International University of Greece, 57001 Thessaloniki, Greece; akontoge@ihu.gr

**Keywords:** Generation Z, eating behavior clusters, emotional eating sensory cues, satiety regulation, factorial and cluster analysis, public health interventions

## Abstract

Background: Building on our previous systematic review that synthesized eight core sustainable appetitive traits central to food behavior research, the present study extends this framework through an empirical investigation of Generation Z university students in Greece. We have established the conceptual foundation by mapping emotional, sensory, and behavioral regulation drivers of eating behavior, underscoring their relevance for nutrition and sustainability. However, empirical applications of this multidimensional framework to Generation Z remained scarce. Objectives: This study addresses this gap by examining eating behaviors among approximately 800 students at the University of Ioannina using a validated post-pandemic questionnaire. Methods & Results: Results revealed heterogeneity across six domains, with consensus observed only in sensory-driven eating (M = 3.88) and openness to new foods (M = 4.00). Cluster analysis identified two distinct profiles: Exploratory and Hedonic Responders and Emotionally Regulated and Satiety-Oriented Responders. These clusters delineate a novel profile of Generation Z, portraying them as digitally immersed, sustainability-oriented, and emotionally sensitive, yet divided between impulsive exploration and regulated satiety. Conclusions: The study contributes new empirical insights into post-pandemic food behavior. It establishes a comprehensive evidence base for designing culturally sensitive wellness programs and targeted nutritional interventions that support sustainable dietary practices. The continuity between the two papers underscores both theoretical importance and the practical necessity of integrating emotional, sensory, and regulatory dimensions in advancing sustainable eating futures among young adults.

## 1. Introduction

The global health crisis of the early 2020s profoundly reshaped global food behavior, altering consumption patterns, emotional responses, and sensory experiences across populations. Among young adults, particularly college students, these changes were especially pronounced due to their transitional developmental stage, digital immersion, and heightened sensitivity to emotional and sensory stimuli [1,2].

Young adulthood represents a developmental stage characterized by heightened susceptibility to disruptions in eating patterns, as biological maturation, identity formation, and psychosocial stressors converge. Recent evidence shows that young adults are particularly vulnerable to disordered eating symptoms, emotional distress, and weight-related health concerns, especially when academic pressure and pervasive digital exposure coexist [3]. Social media environments intensify body image concerns and social comparison processes, which have been linked to emotional overeating, emotional undereating, restrictive tendencies, and other maladaptive eating behaviors in Generation Z [4]. Furthermore, stress-related coping mechanisms involving food have been associated with increased anxiety and depressive symptoms in university populations [5]. Understanding these vulnerabilities is essential, as maladaptive patterns established during young adulthood may persist into later life and contribute to long-term physical and psychological health outcomes. In this context, Generation Z constitutes a critical group for investigation, given their unique psychosocial profile and the rapid sociocultural shifts shaping their dietary experiences.

Generation Z, defined as individuals born between 1995 and 2010, represents a uniquely positioned demographic at the intersection of academic pressures, social transitions, and identity formation. Their eating behaviors provide critical insights into post-pandemic nutritional challenges and opportunities [6,7].

**Figure 1:**
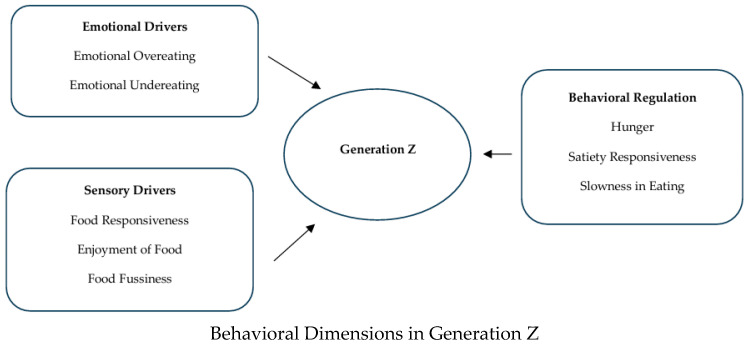
Conceptual flow diagram grouping the eight behavioral factors into three overarching categories, with Generation Z at the center as the focal group.

We identified eight behavioral dimensions in our recent systematic review [8], as central to sustainable food behavior research, namely, the following: Hunger, Food Responsiveness, Emotional Overeating, Enjoyment of Food, Satiety Responsiveness, Emotional Undereating, Food Fussiness, and Slowness in Eating. Building. On this framework, the present study applies these dimensions to Generation Z students in Greece, thereby extending the review’s insights into a specific demographic context and addressing an important research gap. While prior research has examined emotional eating, sustainability, or digital influence separately [9,10,11], few studies have integrated these dimensions into a comprehensive framework for Gen Z food behavior as this study does. The conceptual grouping of the eight factors is visualized in Figure 1, which illustrates their organization into emotional, sensory, and behavioral regulation drivers.

The presentation of the eight behavioral factors in brief is as follows:

1. **Hunger**, which is often perceived cognitively rather than physiologically, with stress and digital distraction influencing its recognition [1,12]. Recent studies show that academic pressure and time constraints can delay eating episodes, reframing hunger as a regulated rather than instinctive cue [13,14].
2. **Food Responsiveness**, which reflects the influence of external sensory stimuli such as sight, smell, and social media exposure. Evidence indicates that Generation Z is particularly sensitive to aesthetic food presentation, which can trigger impulsive consumption [15,16].
3. **Emotional Overeating**, which is a coping mechanism for psychological discomfort, closely linked to anxiety and elevated body mass index. Meta-analyses and recent surveys confirm its relevance in understanding stress-related eating behaviors among young adults [17,18].
4. **Enjoyment of Food**, which is associated with openness to diverse cuisines and social connection. Generation Z demonstrates enthusiasm for culinary exploration, often reinforced by digital food cultures, though this can coexist with weaker satiety awareness [16,19].
5. **Satiety Responsiveness**, which refers to the ability to recognize fullness and regulate intake. Stress and disrupted routines have been shown to weaken internal satiety cues, leading to inconsistent regulation among students [20,21].
6. **Emotional Undereating**, which is characterized by reduced intake under stress or anxiety, has been observed in young adults and remains underexplored in the literature. It represents a coping mechanism that may risk nutritional deficiencies [22,23].
7. **Food Fussiness**, or reluctance to try new foods, which appears to be declining among Generation Z, who show openness to global cuisines and culinary curiosity. Recent studies highlight both cultural and psychological determinants of food neophobia in this demographic [16,24].
8. **Slowness in Eating**, which has implications for satiety and metabolic health. Faster eating, common among students under stress, is associated with reduced satiety awareness and higher BMI [25,26].

In the present manuscript, the term ‘eating behavior’ is used as an overarching descriptor of the examined constructs, while more specific terms such as ‘appetitive traits’ or ‘sustainable food-related behavior’ are employed only when analytically required to denote particular theoretical or empirical dimensions.

### Research Gap & Contribution

Building on the conceptual framework of eight appetitive traits identified in the recent literature, the present study applies this multidimensional structure to Generation Z university students in Greece. The aim is to empirically examine how emotional, sensory, and regulatory eating drivers cluster within this demographic and to explore their implications for sustainable food-related behaviors [8]. In doing so, it addresses a scientific gap: the lack of integrated analyses that combine emotional, sensory, and behavioral regulation factors within the context of a digitally immersed, sustainability-oriented generation.

By situating the eight behavioral dimensions in the lived experiences of Gen Z, this research contributes novel insights into post-pandemic food behavior.

The present study aims to empirically apply the multidimensional framework of emotional, sensory, and regulatory eating drivers to a large sample of Generation Z university students in Greece. By examining how eight validated appetitive traits cluster within this demographic, we seek to identify distinct eating behavior profiles and explore their implications for sustainable food-related practices. In doing so, this work extends our previous systematic review into a concrete post-pandemic context and provides an integrated empirical basis for future public health and nutrition interventions targeting young adults.

## 2. Materials and Methods

### 2.1. Survey Methodology and Data Collection

The empirical investigation was conducted at the University of Ioannina, located in northwestern Greece in a city of approximately 112,000 residents. The institution comprises seven schools and fifteen academic departments, with a total student enrollment of nearly 30,000. Due to the widespread phenomenon of inactive enrollment in Greek higher education, the number of actively engaged students is reduced to approximately 60% of the total, corresponding to roughly 18,000 individuals. Within this group, nearly 20% fall outside the Generation Z cohort and were therefore excluded from the survey population. Consequently, the final eligible population consisted of approximately 14,500 active Generation Z students.

A total of 804 students completed the questionnaire through the Google Forms platform. After excluding four submissions with missing responses on key behavioral items, 800 valid questionnaires were retained for the statistical analyses. Authorization in line with GDPR requirements was obtained from the competent university authority, ensuring that all submissions remained fully anonymous.

This study employed a structured questionnaire to investigate university students’ attitudes toward food-related behaviors. The instrument comprised two main sections. The first section collected sociodemographic information, including gender, age, marital status, employment situation, and place of residence. The second section assessed eight internationally validated appetitive traits: Hunger (H, 5 items), Food Responsiveness (FR, 4 items), Emotional Overeating (EOE, 4 items), Enjoyment of Food (EF, 5 items), Satiety Responsiveness (SR, 5 items), Emotional Undereating (EUE, 5 items), Food Fussiness (FF, 3 items), and Slowness in Eating (SE, 4 items).

These appetitive traits originate from internationally validated instruments, including the Adult Eating Behaviour Questionnaire (AEBQ) and the Child Eating Behaviour Questionnaire (CEBQ), which have been widely applied across diverse populations [27,28]. Although full psychometric validation of the AEBQ in Greece has not yet been published, the instrument has been previously used in Greek samples. In the present study, a forward–backward translation procedure was applied, and all subscales demonstrated satisfactory internal consistency (Cronbach’s α > 0.70), supporting their reliability in this population. The questionnaire was translated into Greek using a forward–backward translation procedure, and all subscales demonstrated satisfactory internal consistency in the present sample, supporting its reliability for use with Greek university students.

Beyond sociodemographic characteristics, these traits were identified through the literature review as key determinants shaping consumers’—and particularly young consumers’—attitudes toward eating behavior. The complete questionnaire is provided in Supplementary Materials as Table S1.

**Inclusion and Exclusion Criteria:** Participation in the study was voluntary and restricted to active university students who belonged to the Generation Z cohort (born 1995–2010) and were enrolled at the University of Ioannina during the data collection period. Only fully complete questionnaires were included in the analysis. Students who reported being older than the Generation Z age range were excluded. No clinical exclusion criteria were applied, as the study did not involve diagnostic assessment; however, participants were asked to confirm that they did not have a current medically diagnosed eating disorder. The questionnaire did not include a formal screening tool for eating disorder risk, consistent with the non-clinical, behavioral focus of the study. This approach ensured broad participation while maintaining alignment with the study’s aims and ethical requirements. Participants who did not report their exact age (*n* = 13) were retained because they provided complete behavioral data and were active university students, a population that falls almost exclusively within the Generation Z age range. Age was not used as an input variable in any statistical procedure (PCA, clustering, or regression), and therefore the inclusion of participants with missing age information does not influence the analytical outcomes.

### 2.2. Analytical Procedures

All items evaluating food-related attitudes were assessed using a five-point Likert scale, ranging from “*Strongly disagree*” (1) to “*Strongly agree*” (5). Descriptive statistics were calculated for each variable, and measures of central tendency were expressed through the mean Likert score. This approach allowed for a more refined depiction of participants’ attitudes, extending beyond simple frequency distributions [29,30].

Given the large sample size (N = 800), mean values were used as summary measures for Likert-type items under the assumption of approximate normality, in line with common practice in behavioral and nutritional research. Both mean values and standard deviations are now reported for all items. This practice is well supported in the literature, as Likert-type responses approximate interval-level properties in large samples, allowing the use of means and standard deviations as valid summary statistics [31,32,33]. Means and standard deviations were reported for descriptive purposes, consistent with Nutrients guidelines and established practice for large-sample Likert data.

To identify latent structures within the twenty-four perception-related Likert items, a principal component analysis (PCA) with Varimax rotation was conducted. PCA was applied as a dimensionality reduction method, converting the initial set of intercorrelated variables into a smaller number of orthogonal components, each reflecting an underlying dimension of sustainability-related attitudes [34]. The adequacy of the dataset for PCA was confirmed through the Kaiser–Meyer–Olkin (KMO) statistic and Bartlett’s test of sphericity [35]. Components with eigenvalues exceeding 1.0 were retained, and only items with factor loadings greater than 0.50 were included in the final solution. The internal consistency of each extracted factor was evaluated using Cronbach’s alpha, with all coefficients surpassing the recommended threshold of 0.70 [36].

Dominant patterns were identified based on standardized factor scores exceeding ±0.50, a threshold commonly used to indicate meaningful deviation from the sample mean in attitudinal research.

The factor scores obtained from the principal component analysis (PCA) were subsequently employed as inputs for clustering procedures. For the clustering procedures, we used the PCA-derived behavioral variables with the highest loadings (the table in Section 3.4) rather than the full set of factor scores. This approach was selected to enhance interpretability and to align the clustering solution with the conceptual structure of the eight appetitive traits. A hierarchical clustering analysis (Ward’s method) was first conducted, and the resulting dendrogram (Supplementary Figure S1) supported a parsimonious two-cluster solution, which was subsequently validated through k-means clustering. For interpretability, the table in Section 3.4 presents the items with the highest loadings on each PCA component, although the clustering procedure itself was performed using the standardized PCA factor scores. Between-cluster differences on the PCA-derived behavioral dimensions showed small-to-large effect sizes (Cohen’s d range: 0.13–1.75), as reported in Supplementary Table S5. The standardized factor scores derived from the PCA were used as input variables in the clustering procedures to avoid multicollinearity and redundancy among highly correlated items. The number of clusters was determined by inspecting the agglomeration schedule and dendrogram, which showed a clear increase in fusion coefficients at the transition from the two- to three-cluster solution, supporting a two-cluster structure. To identify the appropriate number of clusters, a hierarchical clustering approach was first applied, using Ward’s linkage method in combination with squared Euclidean distances. The agglomeration schedule and dendrogram were examined to guide this decision. Inspection of the agglomeration schedule and the dendrogram indicated a clear separation into two major clusters, and alternative K-means solutions (two-, three-, and four-cluster models) were compared, with the two-cluster solution offering the most parsimonious and interpretable structure. The dendrogram illustrating the hierarchical clustering structure is provided in Supplementary Figure S1. Thereafter, a k-means clustering analysis was performed to allocate participants into distinct, non-overlapping, and internally consistent consumer groups [35]. Each cluster reflected a unique attitudinal profile toward sustainable packaging. Differences across clusters were evaluated by comparing the mean values of the PCA-derived dimensions. In the final stage, a binary logistic regression analysis was conducted to determine which factors significantly predicted cluster assignment. Cluster membership was dichotomized according to a median split of the overall behavioral intention scale (coded as 1 = high intention, 0 = low-to-moderate). Predictor variables included all PCA components alongside key sociodemographic characteristics (gender, age category, and educational attainment). Model adequacy was assessed using the Hosmer–Lemeshow goodness-of-fit test, while odds ratios were interpreted through Exp(B) coefficients and their 95% confidence intervals [36]. The logistic regression model demonstrated an overall classification accuracy of 81.2%, compared with a baseline accuracy of 79.5%, indicating a modest but meaningful improvement in discriminative performance. The baseline accuracy corresponds to the initial classification rate obtained from the cluster solution (Step 1), prior to model refinement. The lower accuracy observed for Cluster 2 may reflect greater behavioral heterogeneity or overlapping response patterns, which can reduce classification precision in emotionally driven profiles. All statistical procedures were carried out with IBM SPSS Statistics for Windows, Version 25.0 (IBM Corp., Armonk, NY, USA). No cases were excluded, thereby preserving the completeness and integrity of the dataset.

## 3. Results

### 3.1. Sociodemographic Characteristics

A total of 800 Generation Z students participated in the study. Most respondents identified as female (73.6%), followed by males (23.8%) and a small proportion who did not report gender (2.5%). The majority were aged 21–25 years (57.9%), with 31.8% aged 18–20 and 8.8% aged 26–30. Regarding occupational status, most participants were exclusively students (67.9%), while 31.6% combined studies with employment. Missing demographic data was minimal and did not affect the analytic sample. Sociodemographic characteristics are summarized in Table 1.

### 3.2. Descriptive Statistics of Eating-Behavior Dimensions

Descriptive statistics for the eight validated eating-behavior dimensions are summarized in Table 2. Overall, participants reported moderate levels of hunger cues and food responsiveness, while emotional undereating showed higher mean values than emotional overeating, indicating that negative emotions were more often associated with reduced intake. Satiety responsiveness and enjoyment of food demonstrated consistently high scores, reflecting openness to food variety and positive engagement with eating. Slowness in eating showed moderate values, suggesting variability in self-perceived eating pace. Full item-level descriptive statistics are provided in Supplementary Table S2.

### 3.3. Principal Component Analysis

A principal component analysis (PCA) with Varimax rotation was conducted to identify latent dimensions underlying the 35 eating behavior items. Sampling adequacy was confirmed by a Kaiser–Meyer–Olkin (KMO) value of 0.859 and a significant Bartlett’s test of sphericity (χ^2^ = 15,733.504, *p* < 0.001). Nine components with eigenvalues greater than 1.0 were extracted, explaining 69.7% of the total variance. Items with factor loadings ≥0.40 were retained. Table 3 presents the factor loadings and communalities for all included items. A complete mapping of all items to their corresponding PCA-derived factors is provided in Supplementary Table S3.

### 3.4. Cluster Analysis

**Figure 2:**
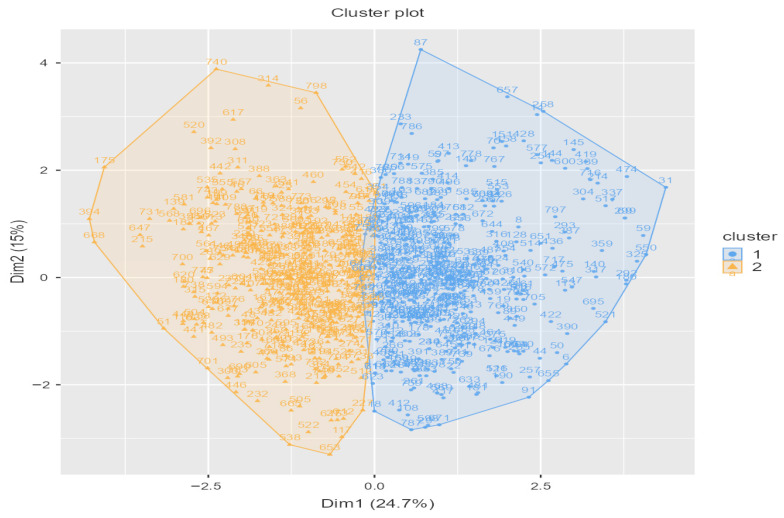
Cluster Plot.

Hierarchical cluster analysis (Ward’s method, squared Euclidean distance) was first applied to determine the optimal number of clusters. Inspection of the agglomeration schedule and the dendrogram (Supplementary Figure S1) indicated a clear increase in fusion distance, supporting a two-cluster solution. A K-means cluster analysis was subsequently performed using the most discriminative items (highest PCA loadings). Table 4 presents the mean values of these items across the two clusters. These mean differences are descriptive; inferential significance was evaluated in the subsequent binary logistic regression model, where the PCA-derived dimensions were included as predictors of cluster membership. The two-dimensional cluster plot is shown in Figure 2. A detailed item-level comparison of the two clusters is provided in Supplementary Table S4, which presents the mean scores of the most discriminative items used in the K-means solution.

Based on the dominant behavioral patterns observed in the discriminative items, the two clusters were conceptually labeled to reflect their underlying profiles. Cluster 1 was characterized by higher enjoyment of food, openness to new foods, and stronger sensory-driven tendencies, and was therefore termed ‘*Exploratory and Hedonic Responders*.’ In contrast, Cluster 2 showed greater emotional regulation, higher satiety responsiveness, and more structured eating patterns, leading to the label ‘*Emotionally Regulated and Satiety-Oriented Responders*.’ These conceptual labels are used throughout the manuscript to facilitate interpretation of the emerging typology

Between-cluster comparisons indicated statistically meaningful differences across all PCA-derived behavioral dimensions, with effect sizes ranging from small to large (Cohen’s d = 0.13–1.75), as shown in Supplementary Table S5.

### 3.5. Logistic Regression

A binomial logistic regression was conducted to identify the most significant predictors of cluster membership. Only variables that reached statistical significance were retained in the final model. Table 5 presents the regression coefficients (B), standard errors, Wald statistics, significance levels, and odds ratios (Exp(B)) for the significant predictors. The logistic regression model demonstrated an overall classification accuracy of 81.2%, compared with a baseline accuracy of 79.5%, indicating a modest but meaningful improvement in discriminative performance. To contextualize this modest improvement in classification accuracy, effect sizes for between-cluster differences are provided in Supplementary Table S5.

## 4. Discussion

The present study examined the multidimensional structure of eating behavior among Generation Z university students and identified distinct behavioral profiles through PCA, cluster analysis, and logistic regression. The findings revealed a coherent yet heterogeneous pattern of eating tendencies, highlighting the interplay of emotional, cognitive, behavioral, and physiological processes in shaping food-related responses in young adults.

Recent studies among university students have similarly reported elevated emotional eating tendencies, particularly in response to academic stress and digital overload [22,37]. Research in young adult populations shows that emotional overeating is positively associated with anxiety symptoms and perceived stress, whereas emotional undereating often emerges as a coping mechanism during periods of heightened psychological strain. These patterns align with our findings, where emotional undereating exceeded emotional overeating, suggesting that negative effect may suppress rather than stimulate intake in a substantial subset of Generation Z students.

Our observation of moderate food responsiveness is consistent with previous work indicating that Generation Z is highly sensitive to external food cues, including visual stimuli and social media exposure [38]. Studies have shown that digital food environments amplify impulsive eating tendencies and increase responsiveness to palatable foods, particularly among young adults who spend extensive time on image-based platforms. This supports the sensory-driven tendencies identified in our sample.

The relatively high satiety responsiveness observed in our study aligns with evidence that young adults can recognize internal fullness cues when not experiencing acute stress [39]. However, prior research also indicates that academic pressure and irregular routines may weaken satiety regulation in university populations [40]. The variability in our sample reflects this duality, suggesting that while many Gen Z students maintain awareness of satiety signals, others may experience disrupted regulatory patterns due to lifestyle instability.

Our findings regarding openness to new foods are consistent with international studies showing that Generation Z demonstrates lower food neophobia and greater culinary curiosity compared with older cohorts [41]. This trend has been attributed to globalization, digital exposure to diverse cuisines, and increased interest in experiential eating. The high scores in enjoyment of food and willingness to try new foods in our sample reinforce this broader generational pattern.

### 4.1. Interpretation of PCA Components

The PCA yielded nine components capturing a broad spectrum of eating-behavior dimensions, including emotional overeating, emotional undereating, food neophobia versus interest, food enjoyment, satiety responsiveness, hunger sensations, and eating pace. This multidimensional structure aligns with contemporary models emphasizing that eating behavior is shaped by both internal regulatory mechanisms and external sensory or cognitive influences. The emergence of distinct emotional and physiological components underscores the importance of affective states and internal cues in shaping eating patterns during young adulthood.

### 4.2. Interpretation of Cluster Profiles

Cluster analysis demonstrated that these components translated into two empirically distinct participant profiles. Cluster 1 reflected a more hedonic and exploratory eating style, characterized by higher enjoyment of food, openness to trying new foods, and stronger hunger-related responsiveness. In contrast, Cluster 2 aligned with an emotionally regulated and satiety-oriented pattern, with higher scores on emotional overeating and satiety responsiveness. Although differences between clusters were moderate, they revealed meaningful distinctions in how young adults engage with food, supporting the heterogeneity of eating behavior within this population.

These cluster patterns are consistent with previous research using the AEBQ and CEBQ frameworks, which similarly identify hedonic–exploratory and regulated–satiety-oriented eating tendencies in young adults. Studies by Hunot et al. [27,28] have shown that enjoyment of food, food responsiveness, and openness to novelty cluster together as markers of exploratory eating, whereas emotional regulation and satiety responsiveness form a distinct behavioral profile. Comparable findings have also been reported in cross-cultural studies examining appetitive traits and their association with eating styles [42]. The present results therefore align with established evidence and extend these patterns to a Greek Generation Z population.

### 4.3. Integration with the Dual Framework

The dual framework derived from the PCA—Group A (emotional and physiological drivers) and Group B (cognitive and behavioral dimensions)—showed clear conceptual alignment with the empirical clusters. Group A corresponded closely to Cluster 2, reflecting affective regulation and satiety-driven processes. Group B aligned with Cluster 1, capturing openness to novelty, hedonic engagement, and responsiveness to external cues. This convergence reinforces the dual nature of internal versus external drivers of eating behavior and illustrates how these mechanisms manifest in lived eating patterns among Generation Z students.

### 4.4. Emerging Typology of Eating Behavior

Integrating the PCA components and cluster profiles yielded two overarching participant types. The first type, corresponding to Cluster 1, reflects a pattern of heightened enjoyment of food, curiosity, openness to food variety, and responsiveness to external cues. The second type, corresponding to Cluster 2, is characterized by eating patterns shaped by emotional states, satiety signals, and heightened physiological sensitivity. This typology provides a refined lens for understanding the interplay of cognitive, behavioral, emotional, and physiological influences on eating behavior and offers a structured framework for interpreting individual differences within the Generation Z cohort.

The two clusters identified in this study—‘Exploratory and Hedonic Responders’ and ‘Emotionally Regulated and Satiety-Oriented Responders’—represent distinct behavioral orientations within Generation Z. The first group reflects a profile characterized by sensory-driven enjoyment, openness to new foods, and a more impulsive, hedonic engagement with eating. In contrast, the second group demonstrates stronger emotional regulation, heightened satiety awareness, and more structured eating patterns. These profiles align with emerging evidence on the heterogeneity of post-pandemic eating behavior among young adults and provide a meaningful framework for interpreting how emotional, sensory, and regulatory drivers coalesce into distinct behavioral typologies.

The conceptual labels assigned to the two clusters were derived from the dominant behavioral patterns observed in the most discriminative items (Supplementary Table S4). These labels do not represent statistical outputs per se, but rather interpretive descriptors intended to facilitate the theoretical integration and practical understanding of the emerging typology.

This typology may support the development of tailored nutritional strategies and sustainability-oriented interventions that account for the emotional and sensory diversity observed within Generation Z.

### 4.5. Predictors of Cluster Membership

The logistic regression analysis identified specific behavioral indicators that significantly predicted cluster membership. Negative coefficients for satiety responsiveness (Q3.4), hunger sensations (Q1.3), physical symptoms of hunger (Q1.4), social eating cues (Q2.3), and food enjoyment (Q7.1) indicated that higher scores on these dimensions decreased the likelihood of belonging to Cluster 2. Conversely, positive coefficients for leaving food unfinished (Q3.3) and initial reluctance toward novel foods (Q5.1) increased the probability of membership in Cluster 2. These findings provide robust statistical support for the multidimensional framework established through the PCA and clustering analyses and highlight the behavioral markers that differentiate hedonic-exploratory from emotionally regulated eating styles.

### 4.6. Broader Context and Implications

Recent international studies indicate that Generation Z exhibits a dual pattern of food engagement, combining strong sensory-driven curiosity with heightened emotional sensitivity [16,43]. This aligns with the present findings, where hedonic–exploratory tendencies coexisted with more regulated, satiety-oriented patterns. Moreover, research on sustainable food acceptance suggests that food neophilia, enjoyment of food, and openness to novelty are positively associated with willingness to adopt environmentally oriented dietary choices [44]. These parallels situate the emerging typology within broader behavioral and sustainability-related frameworks and highlight its relevance for understanding young adults’ eating patterns.

The coexistence of hedonic exploration and regulated satiety highlights the complexity of promoting balanced eating among young adults. Openness to diverse cuisines and sensory curiosity may facilitate the adoption of healthier dietary patterns, whereas emotional suppression and reliance on satiety cues may risk nutritional imbalance if not supported by mindful eating strategies. These findings suggest that interventions should be tailored to address both impulsive and regulated tendencies, integrating emotional support with sensory-based education to foster balanced dietary practices. Comparable approaches have been recommended in recent international studies examining post-pandemic eating behavior among Generation Z populations.

### 4.7. Limitations

This study relied on self-reported data, which may be subject to social desirability bias. The sample was drawn from a single institutional context, potentially limiting generalizability. Additionally, sustainable eating behaviors were not directly measured; therefore, any interpretations related to sustainability should be considered theoretical. Future research should extend this framework to diverse cultural settings and longitudinal designs to capture evolving trajectories of food behavior.

## 5. Conclusions

This study identified a multidimensional structure of eating behavior among Generation Z university students and revealed two distinct behavioral profiles. The first profile (“Hedonic Explorers”) was characterized by higher enjoyment of food, openness to novelty, and stronger responsiveness to external sensory cues. The second profile (“Emotionally Regulated and Satiety-Oriented Responders”) showed greater emotional control, heightened satiety responsiveness, and more structured eating patterns. These profiles emerged consistently across PCA, cluster analysis, and logistic regression, confirming the heterogeneity of eating tendencies within this population.

Key behavioral dimensions—including emotional undereating, moderate food responsiveness influenced by digital cues, high but variable satiety responsiveness, and low food neophobia—collectively illustrate how emotional, sensory, and regulatory mechanisms shape eating patterns in young adults. The coexistence of exploratory–hedonic and emotionally regulated–satiety-oriented tendencies highlights the dual nature of Generation Z’s engagement with food.

These findings provide a clear empirical basis for developing tailored nutritional strategies that address both sensory-driven and emotionally driven eating patterns. Future research should extend this framework to diverse cultural contexts and longitudinal designs to capture evolving trajectories of eating behavior in emerging adulthood.

## Figures and Tables

**Table 1:** Sociodemographic characteristics of the sample.

Characteristics	N	Percentage
	**Gender**	
Male	191	23.8
Female	591	73.6
None of the Above	20	2.5
	**Age**	
18–20	255	31.8
21–25	465	57.9
26–30	71	8.8
Missing	13	1.6
	**Working or not**	
Exclusively Student	545	67.9
Working Student	254	31.6

Note: Totals for each sociodemographic variable may not sum to N = 800 due to item-level missing responses. All 800 participants provided complete data for the behavioral items included in the analysis.

**Table 2:** Summary of Eating-Behavior Dimensions (Mean and SD).

Eating Behavior Dimension	Mean	SD
Hunger (H)	2.58	1.07
Food Responsiveness (FR)	3.01	1.04
Emotional Overeating (EOE)	2.82	1.09
Emotional Undereating (EUE)	3.36	1.22
Satiety Responsiveness (SR)	3.39	1.06
Enjoyment of Food (EF)	3.86	0.86
Slowness in Eating (SE)	2.85	1.17

Note: Food Fussiness items (Q5.1–Q5.5) were conceptually integrated into Satiety Responsiveness, consistent with the PCA structure and theoretical grouping.

**Table 3:** Principal Component Analysis—Factor Loadings and Communalities.

Properties	Communalities	Fact 1	Fact 2	Fact 3	Fact 4	Fact 5	Fact 6	Fact 7	Fact 8	Fact 9
Q4.3 I eat less when I’m anxious	0.884	0.898								
Q4.2 I eat less when I’m worried	0.862	0.884								
Q4.4 I eat less when I’m upset	0.792	0.739							0.445	
Q6.2 I eat more when I’m worried	0.838	−0.738				0.494				
Q6.3 I eat more when I’m anxious	0.845	−0.734				0.513				
Q5.4 I enjoy tasting new foods	0.807		0.884							
Q5.2 I am interested in tasting new food I haven’t tasted before	0.771		0.861							
Q5.1 I refuse new foods at first	0.746		−0.849							
Q5.3 I often decide that I don’t like a food, before tasting it	0.639		−0.76							
Q5.5 I enjoy a wide variety of foods	0.589		0.72							
Q7.2 I love food	0.763			0.826						
Q7.1 I enjoy eating	0.739			0.826						
Q7.3 I look forward to mealtimes	0.637			0.74						
Q2.4 When I see or smell food that I like, it makes me want to eat	0.407			0.475						
Q2.3 I often feel hungry when I am with someone who is eating	0.495			0.455						
Q2.1 I am always thinking about food	0.472			0.423						
Q2.2 Given the choice, I would eat most of the time	0.485									
Q8.2 I am often last at finishing a meal	0.843				0.893					
Q8.1 I eat slowly	0.820				0.888					
Q8.3 I often finish my meals quickly	0.800				−0.869					
Q8.4 I eat more and more slowly during the course of a meal	0.495				0.631					
Q6.5 I eat more when I’m angry	0.874					0.866				
Q6.4 I eat more when I’m annoyed	0.895					0.863				
Q6.1 I eat more when I’m upset	0.764	−0.596				0.609				
Q3.3 I often leave food on my plate at the end of a meal	0.690						0.78			
Q3.4 I often get full before my meal is finished	0.643						0.75			
Q3.1. I get full up easily	0.555						0.659			
Q3.2 I cannot eat a meal if I have had a snack just before	0.438						0.606			
Q1.1 I often notice my stomach rumbling	0.673							0.81		
Q1.3 I often feel hungry	0.653							0.727		
Q1.2 I often feel so hungry that I have to eat something right away	0.612							0.702		
Q4.1 I eat less when I’m annoyed	0.789								0.759	
Q4.5 I eat less when I’m angry	0.787								0.745	
Q1.5 If I miss a meal I get irritable	0.659									0.791
Q1.4 If my meals are delayed I get light-headed	0.633									0.744
Initial Eigenvalues		7.85	3.926	3.216	2.744	1.747	1.431	1.296	1.128	1.057
Percentage of variance explained		11.442	10.071	8.651	8.442	7.896	7.306	5.845	5.087	4.96
Cronbach’s a reliability test						0.698 (35 items)				
Total variance %						69.7%				

Note: Factor loadings greater than 0.4 are presented. Extraction method: principal component analysis rotation method using varimax with Kaiser normalization. Kaiser–Meyer–Olkin (KMO) 0.859; Bartlett’s test of sphericity 0.00 (χ^2^ = 15,733.504).

**Table 4:** Mean values in questions used in cluster analysis for the two clusters.

Question	Mean Value *	
	Cluster 1	Cluster 2
*Q4 [3. I eat less when I am anxious]*	3.37	3.34
*Q5 [4. I enjoy tasting new foods]*	3.94	3.71
*Q7 [2. I love food]*	4.15	3.94
*Q8 [2. I am often last at finishing a meal]*	2.85	2.91
*Q6 [5. I eat more when I am angry]*	2.18	2.45
*Q3 [3. I often leave food on my plate at the end of a meal]*	2.47	2.69
*Q1 [1. I often notice my stomach rumbling]*	3.08	2.94
*Q4 [1. I eat less when I’m annoyed]*	3.10	2.98
*Q1 [5. If I miss a meal I get irritable]*	2.73	2.44

* Note: Values range from one to five.

**Table 5:** Binomial logistic regression results predicting students’ participation in clusters.

Variables	Coefficient B (β)	S.E.	Wald Statistic	Wald Sig.	Exp(B)
Q3.4 I often get full before my meal is finished	−0.305	0.099	9.539	0.002	0.737
Q1.3 I often feel hungry	−0.214	0.092	5.422	0.020	0.808
Q1.4 If my meals are delayed, I get light-headed	−0.183	0.069	7.112	0.008	0.833
Q2.3 I often feel hungry when I am with someone who is eating	−0.160	0.080	4.017	0.045	0.852
Q3.3 I often leave food on my plate at the end of a meal	0.287	0.087	10.819	0.001	1.333
Q5.1 I refuse new foods at first	0.181	0.077	5.473	0.019	1.199
Q7.1 I enjoy eating	−0.249	0.106	5.559	0.018	0.780
Constant	0.359	0.084	18.445	<0.001	1.432

Note: R^2^ = 0.324 (Nagelkerke), All predictor variables were treated as single-item Likert-type scale items, measured on a 5-point agreement scale (1 = strongly disagree, 5 = strongly agree). The odds ratios (Exp(B)) represent the change in odds of supporting sustainable packaging for each unit increase in agreement with the corresponding statement.

## Data Availability

The data presented in this study is available on request from the corresponding author due to the fact that is part of an ongoing study.

## Supplementary Materials

The following supporting information can be downloaded at: https://www.mdpi.com/article/10.3390/nu18050758/s1, Table S1: Questionnaire on Consumer Dietary Behavior in the Post-Covid Era; Table S2: Full item-level descriptive statistics (Mean and SD) for all questionnaire items across the eight eating-behavior dimensions; Table S3: Mapping of questionnaire items to the nine PCA derived factors; Table S4: Mean scores of the most discriminative items used in the K means cluster analysis among Generation Z students at the University of Ioannina; Table S5: Mean scores and standard deviations of the eight psycho nutritional dimensions across the two clusters; Figure S1: The dendrogram illustrates the hierarchical structure of the sample based on the PCA derived behavioral variables with the highest loadings (Table 4). The clear increase in fusion coefficients at the transition from the two to three cluster solution supports the selection of a parsimonious two cluster structure, which was subsequently validated through k means clustering.
