# Supplementary material for: From Traits to Clusters: Emotional–Sensory–Regulatory Eating Profiles in Generation Z with Implications in Sustainable Food Behavior
Source: Nutrients. 2026 Feb 26;18(5):758. doi: 10.3390/nu18050758 (PMC12986968; doi:10.3390/nu18050758)

nutrients

MDPI

From Traits to Clusters: Emotional–Sensory–Regulatory Eating Profiles in Generation Z with Implications in Sustainable Food Behavior

Maria P. Koliou [1], Amalia Kouskoura [2]Achilleas Kontogeorgos [3], Dimitris Skalkos [1*]

[1] Laboratory of Food Chemistry, Department of Chemistry, University of Ioannina, 45110 Ioannina, Greece; m.koliou@uoi.gr (MPK)
[2] Management of Technology Research Lab (Materlab), University of Western Macedonia, 50100 Kozani, Greece, akouskoura@uowm.gr (A.K.)
[3] Department of Agriculture, International University of Greece, 57001 Thessaloniki, Greece; akontoge@ihu.gr (Ac.K)
* Correspondence: dskalkos@uoi.gr ; Tel.: +30-2651008345

Supplementary material

Table S1

Questionnaire on Consumer Dietary Behavior in the Post-Covid Era.

I. DEMOGRAPHICS

**1. SEX**
MALE
FEMALE
OTHER
I PREFER NOT TO MENTION

**2. Age**
18-20
20-25
25-30
30-35

**3. Civil state**
Single
Married
Divorced

**4. Job situation**
Employed student
Student exclusively

**5. Residency**
NORTH GREECE (regions of Macedonia – Thrace)
WEST GREECE (region of Epirus – Aitoloakarnania prefecture)
CENTRAL GREECE (including Athens)
SOUTH GREECE (region of Peloponnese)
ISLANDS

Choose in the scale between Strongly disagree "1" to Strongly Agree "5" where "1" represents a **STRONG OPPOSITION TO THE STATEMENT** and "5" represents a **FULL AGREE- IDENTIFICATION WITH THE STATEMENT. The "3" represents the NEUTRAL RESPONSE DUE TO LACK OF CERTAINTY AND DOUBTS.**

(Mark your answer with X).

Appetitive Traits

1. Hunger (H)

Compared to usual	Strongly Disagree	Disagree	Neither Agree OR Disagree	Agree	Strongly Agree
1. I often notice my stomach rumbling.	☐ 1	☐ 2	☐ 3	☐ 4	☐ 5
2. I often feel so hungry that I have to eat something right away.	☐ 1	☐ 2	☐ 3	☐ 4	☐ 5
3. I often feel hungry	☐ 1	☐ 2	☐ 3	☐ 4	☐ 5
4. If my meals are delayed I get light-headed	☐ 1	☐ 2	☐ 3	☐ 4	☐ 5
5. If I miss a meal I get irritable	☐ 1	☐ 2	☐ 3	☐ 4	☐ 5

2. Food Responsiveness (FR)

Compared to usual	Strongly Disagree	Disagree	Neither Agree OR Disagree	Agree	Strongly Agree
1. I am always thinking about food	☐ 1	☐ 2	☐ 3	☐ 4	☐ 5
2. Given the choice, I would eat most of the time	☐ 1	☐ 2	☐ 3	☐ 4	☐ 5
3. I often feel hungry when I am with someone who is eating	☐ 1	☐ 2	☐ 3	☐ 4	☐ 5
4. When I see or smell food that I like, it makes me want to eat	☐ 1	☐ 2	☐ 3	☐ 4	☐ 5

3. Emotional Over Eating (EOE)

Compared to usual	Strongly Disagree	Disagree	Neither Agree OR Disagree	Agree	Strongly Agree
1. I get full up easily	☐ 1	☐ 2	☐ 3	☐ 4	☐ 5
2. I cannot eat a meal if I have had a snack just before	☐ 1	☐ 2	☐ 3	☐ 4	☐ 5
3. I often leave food on my plate at the end of a meal	☐ 1	☐ 2	☐ 3	☐ 4	☐ 5
4. I often get full before my meal is finished	☐ 1	☐ 2	☐ 3	☐ 4	☐ 5

4. Enjoyment of food (EF)

Compared to usual	Strongly Disagree	Disagree	Neither Agree OR Disagree	Agree	Strongly Agree
1. I eat less when I'm annoyed	☐ 1	☐ 2	☐ 3	☐ 4	☐ 5
2. I eat less when I'm worried	☐ 1	☐ 2	☐ 3	☐ 4	☐ 5
3. I eat less when I'm anxious	☐ 1	☐ 2	☐ 3	☐ 4	☐ 5
4. I eat less when I'm upset	☐ 1	☐ 2	☐ 3	☐ 4	☐ 5
5. I eat less when I'm angry	☐ 1	☐ 2	☐ 3	☐ 4	☐ 5

5. Satiety Responsiveness (SR)

Compared to usual	Strongly Disagree	Disagree	Neither Agree OR Disagree	Agree	Strongly Agree
1. I refuse new foods at first	☐ 1	☐ 2	☐ 3	☐ 4	☐ 5
2. I am interested in tasting new food I haven't tasted before	☐ 1	☐ 2	☐ 3	☐ 4	☐ 5
3. I often decide that I don't like a food, before tasting it	☐ 1	☐ 2	☐ 3	☐ 4	☐ 5
4. I enjoy tasting new foods	☐ 1	☐ 2	☐ 3	☐ 4	☐ 5
5. I enjoy a wide variety of foods	☐ 1	☐ 2	☐ 3	☐ 4	☐ 5

6. Emotional Under- Eating (EUE)

Compared to usual	Strongly Disagree	Disagree	Neither Agree OR Disagree	Agree	Strongly Agree
1. I eat more when I'm upset	☐ 1	☐ 2	☐ 3	☐ 4	☐ 5
2. I eat more when I'm worried	☐ 1	☐ 2	☐ 3	☐ 4	☐ 5
3. I eat more when I'm anxious	☐ 1	☐ 2	☐ 3	☐ 4	☐ 5
4. I eat more when I'm annoyed	☐ 1	☐ 2	☐ 3	☐ 4	☐ 5
5. I eat more when I'm angry	☐ 1	☐ 2	☐ 3	☐ 4	☐ 5

7. Food Fussiness (FF)

Compared to usual	Strongly Disagree	Disagree	Neither Agree OR Disagree	Agree	Strongly Agree
1. I enjoy eating	☐ 1	☐ 2	☐ 3	☐ 4	☐ 5
2. I love food	☐ 1	☐ 2	☐ 3	☐ 4	☐ 5
3. I look forward to mealtimes	☐ 1	☐ 2	☐ 3	☐ 4	☐ 5

8. Slowness in Eating (SE)

Compared to usual	Strongly Disagree	Disagree	Neither Agree OR Disagree	Agree	Strongly Agree
1. I eat slowly	☐ 1	☐ 2	☐ 3	☐ 4	☐ 5
2. I am often last at finishing a meal	☐ 1	☐ 2	☐ 3	☐ 4	☐ 5
3. I often finish my meals quickly	☐ 1	☐ 2	☐ 3	☐ 4	☐ 5
4. I eat more and more slowly during the course of a meal	☐ 1	☐ 2	☐ 3	☐ 4	☐ 5

Table S2. Full item-level descriptive statistics (Mean and SD) for all questionnaire items across the eight eating-behavior dimensions.

Question Code	Hunger	Mean Value*	Standard Deviation
Q1.1	I often notice my stomach rumbling	2.57	.926
Q1.2	I often feel so hungry that I have to eat something right away.	2.61	1.013
Q1.3	I often feel hungry	2.94	.951
Q1.4	If my meals are delayed, I get light-headed	2.36	1.243
Q1.5	If I miss a meal, I get irritable	2.41	1.203
	**Food Responsiveness**		
Q2.1	I am always thinking about food	2.61	1.099
Q2.2	Given the choice, I would eat most of the time	2.74	1.072
Q2.3	I often feel hungry when I am with someone who is eating	2.79	1.103
Q2.4	When I see or smell food that I like, it makes me want to eat	3.88	.897
	**Emotional Overeating** (items reflecting satiety/fullness cues)		
Q3.1	I get full up easily	3.09	.980
Q3.2	I cannot eat a meal if I have had a snack just before	2.66	1.096
Q3.3	I often leave food on my plate at the end of a meal	2.53	1.215
Q3.4	I often get full before my meal is finished	3.00	1.049
	**Enjoyment of Food** (items reflecting emotional under-eating)		

Q4.1	I eat less when I'm annoyed	3.12	1.173
Q4.2	I eat less when I'm worried	3.47	1.294
Q4.3	I eat less when I'm anxious	3.45	1.277
Q4.4	I eat less when I'm upset	3.45	1.204
Q4.5	I eat less when I'm angry	3.32	1.166
	**Satiety Responsiveness** (items reflecting food neophobia vs. food interest)		
Q5.1	I refuse new foods at first	2.37	1.085
Q5.2	I am interested in tasting new food I haven't tasted before	4.00	1.051
Q5.3	I often decide that I don't like a food, before tasting it	2.73	1.153
Q5.4	I enjoy tasting new foods	3.95	.986
Q5.5	I enjoy a wide variety of foods	3.88	1.031
	**Emotional Under-Eating**		
Q6.1	I eat more when I'm upset	2.33	1.088
Q6.2	I eat more when I'm worried	2.34	1.130
Q6.3	I eat more when I'm anxious	2.34	1.138
Q6.4	I eat more when I'm annoyed	2.26	1.032
Q6.5	I eat more when I'm angry	2.22	1.001
	**Food Fussiness**		
Q7.1	I enjoy eating	4.00	.851
Q7.2	I love food	4.02	.824
Q7.3	I look forward to mealtimes	3.57	.915
	**Slowness in Eating**		
Q8.1	I eat slowly	2.96	1.156
Q8.2	I am often last at finishing a meal	2.88	1.321
Q8.3	I often finish my meals quickly	2.96	1.210
Q8.4	I eat more and more slowly during the course of a meal	2.58	1.001

*Note: Values range from one to five.

Table S3: Mapping of questionnaire items to the nine PCA-derived factors.

Factor	Items (Q)	Description
Factor 1- Emotional Under-Eating	Q4.1, Q4.2, Q4.3, Q4.4, Q4.5	Eating less when anxious, upset, or angry
Factor 2- Food Neophobia vs. Food Interest	Q5.1, Q5.2, Q5.3, Q5.4, Q5.5	Reluctance vs. willingness to try new foods
Factor 3- Food Enjoyment	Q7.1, Q7.2, Q7.3	Pleasure and anticipation of eating
Factor 4- Eating Pace	Q8.1, Q8.2, Q8.3, Q8.4	Slow vs. fast eating tendencies
Factor 5- Emotional Over- Eating	Q6.1, Q6.4, Q6.5	Increased food intake under anger or distress
Factor 6- Satiety Responsiveness	Q3.1, Q3.2, Q3.3, Q3.4	Sensitivity to fullness and meal termination
Factor 7- Hunger Sensations	Q1.1, Q1.2, Q1.3	Physiological hunger signals
Factor 8- Irritability & Physical Symptoms of Hunger	Q1.4, Q1.5	Irritability and dizziness when meals are delayed

Factor 9- Food Responsiveness	Q2.1, Q2.2, Q2.3, Q2.4	Responsiveness to external food cues

Table S4: Mean scores of the most discriminative items used in the K-means cluster analysis among Generation Z students at the University of Ioannina.

Clusters Characteristics	*First Cluster:* "Exploratory and Hedonic Responders"	*Second Cluster:* "Emotionally Regulated and Satiety-Oriented Responders"
	Factor 1	
Q4.2 I eat less when I'm worried	3.35	3.39
Q4.4 I eat less when I'm upset	3.42	3.31
Q6.2 I eat more when I'm worried	2.50	2.43
Q6.3 I eat more when I'm anxious	2.42	2.53
	Factor 2	
Q5.2 I am interested in tasting new food I haven't tasted before *	3.93	3.76
Q5.1 I refuse new foods at first *	2.41	2.61
Q5.3 I often decide that I don't like a food, before tasting it	2.71	2.89
Q5.5 I enjoy a wide variety of foods *	3.74	3.58
	Factor 3	
Q7.1 I enjoy eating *	4.12	3.93
Q7.3 I look forward to mealtimes *	3.58	3.41
Q2.4 When I see or smell food that I like, it makes me want to eat	3.80	3.70
Q2.3 I often feel hungry when I am with someone who is eating *	3.17	2.91
Q2.1 I am always thinking about food *	2.83	2.62
	Factor 4	
Q8.1 I eat slowly	2.89	3.04
Q8.3 I often finish my meals quickly	3.02	2.95
Q8.4 I eat more and more slowly during the course of a meal	2.62	2.65
	Factor 5	
Q6.4 I eat more when I'm annoyed *	2.21	2.48
Q6.1 I eat more when I'm upset *	2.39	2.57
	Factor 6	
Q3.4 I often get full before my meal is finished	3.14	3.13
Q3.1 I get full up easily	3.31	3.41
Q3.2 I cannot eat a meal if I have had a snack just before	2.74	2.76
	Factor 7	
Q1.3 I often feel hungry *	3.29	3.06
Q1.2 I often feel so hungry that I have to eat something right away	2.98	2.89
	Factor 8	
Q4.5 I eat less when I'm angry	3.16	3.02

	Factor 9	
Q1.4 If my meals are delayed I get light-headed	2.60	2.43

* These results are significant statistically different based on one-way anova.

Table S5. Mean scores and standard deviations of the eight psycho-nutritional dimensions across the two clusters.

Dimension	Cluster 1 Mean (SD)	Cluster 2 Mean (SD)	Total Mean (SD)
Q1	2.5781 (0.6658)	3.4252 (0.7640)	2.9713 (0.8286)
Q2	3.2561 (0.7520)	2.9178 (0.7432)	3.0991 (0.7663)
Q3	2.5781 (0.6658)	3.4252 (0.7640)	2.9713 (0.8286)
Q4	2.6167 (0.8348)	3.9462 (0.6783)	3.2338 (1.0132)
Q5	3.3450 (0.3754)	3.2976 (0.3777)	3.3230 (0.3769)
Q6	2.8479 (0.9266)	1.9764 (0.6828)	2.4434 (0.9299)
Q7	4.0477 (0.6652)	3.5754 (0.7464)	3.8285 (0.7420)
Q8	2.6557 (0.4740)	3.1240 (0.5104)	2.8731 (0.5437)

Note. Each psycho-nutritional dimension (Q1–Q8) was computed as the mean score of its corresponding items. The number of items per dimension varied according to the structure of the original instrument: Q1 (5 items: a–e), Q2 (4 items: a–d), Q3 (4 items: a–d), Q4 (5 items: a–e), Q5 (5 items: a–e), Q6 (5 items: a–e), Q7 (3 items: a–c), and Q8 (4 items: a–d). All available items for each dimension were included in the computation of the composite score.

Supplementary Figure S1. The dendrogram illustrates the hierarchical structure of the sample based on the PCA-derived behavioral variables with the highest loadings (Table 4). The clear increase in fusion coefficients at the transition from the two- to three-cluster solution supports the selection of a parsimonious two-cluster structure, which was subsequently validated through k-means clustering.

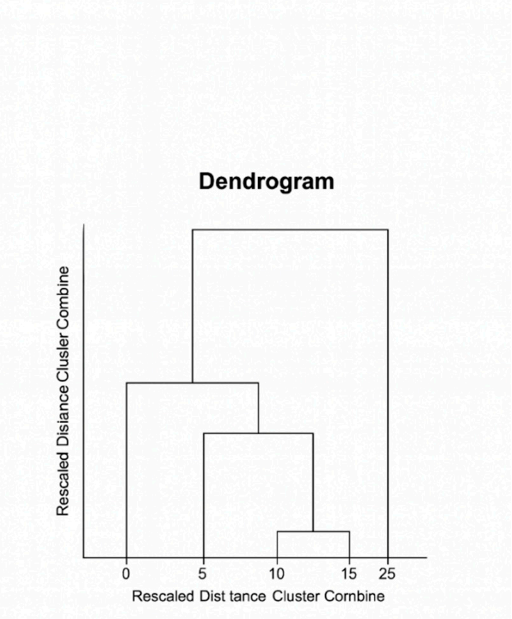

Supplement: Supplementary file 1 [file nutrients-18-00758-s001.zip › nutrients-4164415-supplementary.pdf]
